# Deep convolutional network-based chest radiographs screening model for pneumoconiosis

**DOI:** 10.3389/fmed.2024.1290729

**Published:** 2024-01-29

**Authors:** Xiao Li, Ming Xu, Ziye Yan, Fanbo Xia, Shuqiang Li, Yanlin Zhang, Zhenzhen Xing, Li Guan

**Affiliations:** ^1^Peking University Third Hospital, Beijing, China; ^2^Beijing Tianming Innovation Data Technology Co., Ltd., Beijing, China

**Keywords:** artificial intelligence, pneumoconiosis, convolutional neural network, computer-aided diagnosis, chest radiograph

## Abstract

**Background:**

Pneumoconiosis is the most important occupational disease all over the world, with high prevalence and mortality. At present, the monitoring of workers exposed to dust and the diagnosis of pneumoconiosis rely on manual interpretation of chest radiographs, which is subjective and low efficiency. With the development of artificial intelligence technology, a more objective and efficient computer aided system for pneumoconiosis diagnosis can be realized. Therefore, the present study reported a novel deep learning (DL) artificial intelligence (AI) system for detecting pneumoconiosis in digital frontal chest radiographs, based on which we aimed to provide references for radiologists.

**Methods:**

We annotated 49,872 chest radiographs from patients with pneumoconiosis and workers exposed to dust using a self-developed tool. Next, we used the labeled images to train a convolutional neural network (CNN) algorithm developed for pneumoconiosis screening. Finally, the performance of the trained pneumoconiosis screening model was validated using a validation set containing 495 chest radiographs.

**Results:**

Approximately, 51% (25,435/49,872) of the chest radiographs were labeled as normal. Pneumoconiosis was detected in 49% (24,437/49,872) of the labeled radiographs, among which category-1, category-2, and category-3 pneumoconiosis accounted for 53.1% (12,967/24,437), 20.4% (4,987/24,437), and 26.5% (6,483/24,437) of the patients, respectively. The CNN DL algorithm was trained using these data. The validation set of 495 digital radiography chest radiographs included 261 cases of pneumoconiosis and 234 cases of non-pneumoconiosis. As a result, the accuracy of the AI system for pneumoconiosis identification was 95%, the area under the curve was 94.7%, and the sensitivity was 100%.

**Conclusion:**

DL algorithm based on CNN helped screen pneumoconiosis in the chest radiographs with high performance; thus, it could be suitable for diagnosing pneumoconiosis automatically and improve the efficiency of radiologists.

## 1 Background

Pneumoconiosis is a major pulmonary occupational disease worldwide. Inhaling a solid-phase non-living particulate matter (e.g., inorganic dust) can result in this disease (1). Owing to its irreversible and crippling nature as well as the fatality associated with it, the disease puts a strain on society. Mainly, industrial workers get exposed to exhalable inorganic dust such as asbestos, silica, and coal dust; thus, they are at a higher risk of pneumoconiosis. Over the past few decades, many measures have been taken to protect workers from dust inhalation. However, pneumoconiosis remains a threat to public health. According to the newly published Global Burden of Disease study, there are still a large number of patients. The prevalence of pneumoconiosis is approximately 3,072,550 cases, with 199,125 new cases reported globally in 2019 (2). In recent years, the mortality rate of pneumoconiosis patients has remained at a high level, with 23,015 deaths in 2019 (2). It is important to note that pneumoconiosis is resurgent in the United States and Australia, even though these countries have highly developed healthcare systems, high standards of workplace safety procedures, and highly mechanized mining processes that reduce workers’ exposure to dust (3). It is conceivable that other less developed countries, especially those with poor reporting systems, may have many undiagnosed and unreported cases. Therefore, the prevalence and mortality of pneumoconiosis may be even higher than currently reported.

Despite the heavy social burden of pneumoconiosis, a better prognosis can be achieved by detecting, diagnosing, and treating the disease as early as possible. The International Labor Organization (ILO) guidelines indicate chest radiography as the easiest and the most affordable radiological test for the physical checkup of dust-exposed workers and mass screening of them for the disease (4). However, greater volumes of images produced during the test put a burden on radiologists; thus, limitations such as low efficiency and poor stability are associated with this test. Furthermore, radiographic interpretations are not objective because they depend on radiologists’ work experience. Less-experienced physicians may provide inaccurate interpretations of radiographs, which can lead to misdiagnosis or missed diagnosis. Hence, a computer-aided diagnosis (CAD) strategy has been developed for detecting pneumoconiosis accurately and rapidly. It will help decrease radiologists’ workload and improve their efficiency in mass screening pneumoconiosis using chest radiography techniques. Using this system, a diagnostic standard can be provided for the reference of less-experienced radiologists. Additionally, it will be a useful means for radiological training.

Pneumoconiosis has been detected with CAD systems using texture analysis and hybrid optical–digital methods, including optical Fourier transform, artificial neural networks (ANNs), and support vector machines (SVMs) (5–9). However, these methods might use either non-neural or shallow neural networks, thus requiring further manual extraction and excess time. Particularly, these methods are not suitable for efficient representational learning to achieve maximum accuracy. Conversely, representational learning networks such as deep convolutional neural networks (CNNs) automatically extract task-related features. Hence, compared with traditional methods, deep learning (DL) in auxiliary diagnosis modeling can markedly improve diagnostic accuracy and simultaneously reduce the workload.

DL-based artificial intelligence (AI) systems can expedite and improve image modality interpretations, including interpretations of computed tomography, magnetic resonance, and radiography images, thus advancing diagnostic systems in radiology. Applying DL algorithms to chest radiographs is being assessed for diverse thoracic diseases, including lung nodules (10), pulmonary tuberculosis (11), pneumonia (12), and pneumothorax (13) as well as pectoral abnormities such as pulmonary opacities, pleural effusions, hilar prominence, and enlarged cardiac silhouette (14). Recently, a DL-based algorithm was trained for detecting pulmonary malignant nodules observed in chest radiographs on the basis of a dataset comprising 43,292 images with a normal-to-diseased ratio of 3.67. A comparison between the performances of the algorithm and 18 experienced physicians showed that the area under the curve (AUC) was higher for the DL algorithm than for the 18 physicians. Moreover, for all physicians, nodule detection accuracy was improved in the case of algorithm-assisted diagnosis (15). In another study, two CNN systems were trained using a dataset comprising 1,007 chest radiographs of normal individuals and patients with tuberculosis (492 patients with tuberculosis and 515 normal individuals). Combining the two CNNs, the AUC of a classifier with the best performance was 0.99 (11). In addition to the aforementioned information, DL systems have been used for detecting multiple diseases with promising results (16). Hwang et al. developed a DL algorithm, which was trained using a database comprising normal (*n* = 54,221) and abnormal (*n* = 35,613) cases to differentiate normal chest radiographs from abnormal ones. Active tuberculosis, malignant neoplasms, pneumonia, and pneumothorax were considered abnormal. The median AUC of the algorithm (0.979) was superior to that of radiologists. Nevertheless, these radiologists performed better with the assistance of the AI algorithm (17). These studies show that radiography interpretation is an ideal field for applying automatic interpretations using DL algorithms, and its preliminary practice has yielded promising results and warrants more effort in its progress (18).

Large-scale studies regarding the diagnostic performance of pneumoconiosis by DL algorithms remain scarce and the performance was unsatisfactory. Therefore, we developed a novel CAD tool showing excellent performance using CNNs for the case-based screening of pneumoconiosis on the basis of large-scale chest radiographs, which can help radiologists in interpreting image modalities, thereby improving the efficiency of pneumoconiosis diagnosis and occupational health monitoring for dust exposed workers.

## 2 Methods

### 2.1 Data sources and data screening

Here, we developed a chest radiograph database that comprised more than 482,678 images of digital radiography (DR) from 35 hospitals in 17 provinces of China. A total of 50,367 chest radiographs of patients with pneumoconiosis or workers exposed to dust were chosen from the developed database. Fourth-class, non-posterior–anterior, and non-pneumoconiosis chest radiographs were excluded after data annotations to adhere to the technical requirements for identifying pneumoconiosis, especially the requirement that chest radiographs need to be obtained with the help of posterior–anterior projection as well as DR.

Since there are a large number of negative data in the basic database, the model completed in the last round of training will be used to screen the data, and the most likely positive data will be selected according to the model results. Then, the obtained data were labeled for the subsequent step.

Finally, the chosen chest radiographs were randomized into two datasets in an 8:2 ratio as follows: a training dataset comprising 39,898 chest radiographs (19,705 positive and 20,193 negative ones) was used for the training purpose and a test dataset comprising 9,974 chest radiographs (4,887 positive and 5,087 negative ones) was used for validating network detection performance. Experienced radiologists annotated the test and training datasets in the same way, and the similar positive rates (pneumoconiosis) of the two datasets exceeded 49%. Patients in the two datasets were exclusively different from each other. Images with the pneumoconiosis label were considered positive. Furthermore, images with the other-pulmonary-disease label were not used for training purposes.

This study was approved by the Medical Science Research Ethics Committee of Peking University Third Hospital [Ethics No. (2023) Medical Ethics Review No. (100-01)], all methods were carried out in accordance with relevant guidelines and regulations. The written informed consent to participate was obtained from all the patients.

### 2.2 Data annotations

#### 2.2.1 Annotation tool

We annotated the data using in-house software.^1^ Furthermore, we standardized these annotations on the basis of self-formulated process regulations.

#### 2.2.2 Annotation method and reference standard

Ten experts in diagnosing occupational diseases and two professional radiologists annotated the chest radiographs assessed in the present study. These experts and radiologists were blinded to each other’s evaluations.

Each radiograph had three–five annotated records that contained thorough information regarding the following: chest radiograph quality (first-class, second-class, third-class, and fourth-class), pneumoconiosis opacity characteristics (size, shape, distribution, and location), and diagnosis (normal, pneumoconiosis, and other pulmonary diseases). Based on the ILO guidelines (4), each lung field was divided into the following three zones: upper, middle, and lower. Pneumoconiosis was diagnosed on the basis of the shape, size, and profusion of the opacities of pneumoconiosis (Table 1). Profusion levels of small opacities indicated their concentrations in damaged lung zones, thus reflecting pneumoconiosis degree. Profusion was identified by comparing standard radiographs and denoted as categories 0, 1, 2, and 3 as well as subcategories 0/−, 0/0, 0/1, 1/0, 1/1, 1/2, 2/1, 2/2, 2/3, 3/2, 3/3, and 3/+. Small opacities were absent or small opacities with less profuse were present in category 0 (normal individuals) compared with those in category 1, whereas category 3 indicated the most severe cases. The following additional codes were used during annotation to identify other pulmonary diseases: bu—pneumatocele, ca—lung cancer and mesothelioma of pleura, cn—small opacity calcification, cp—pulmonary heart disease, cv—pulmonary cavity, em—emphysema, ef—pleural effusion, es—eggshell-like calcification in lymph nodes, ho—honeycomb lung, pc—pleural calcification, pt—pleural thickening, rp—rheumatic pneumoconiosis, px—pneumothorax, and tb—active tuberculosis.

**Table 1:** Classification of small opacities.

Rounded opacities	*p*	Diameter up to 1.5 mm
	*q*	Diameter 1.5–3 mm
	*r*	Diameter 3–10 mm
Irregular opacities	*s*	Width up to 1.5 mm
	*t*	Width 1.5–3 mm
	*u*	Width 3–10 mm

#### 2.2.3 Annotation quality control algorithm

**Figure 1:**
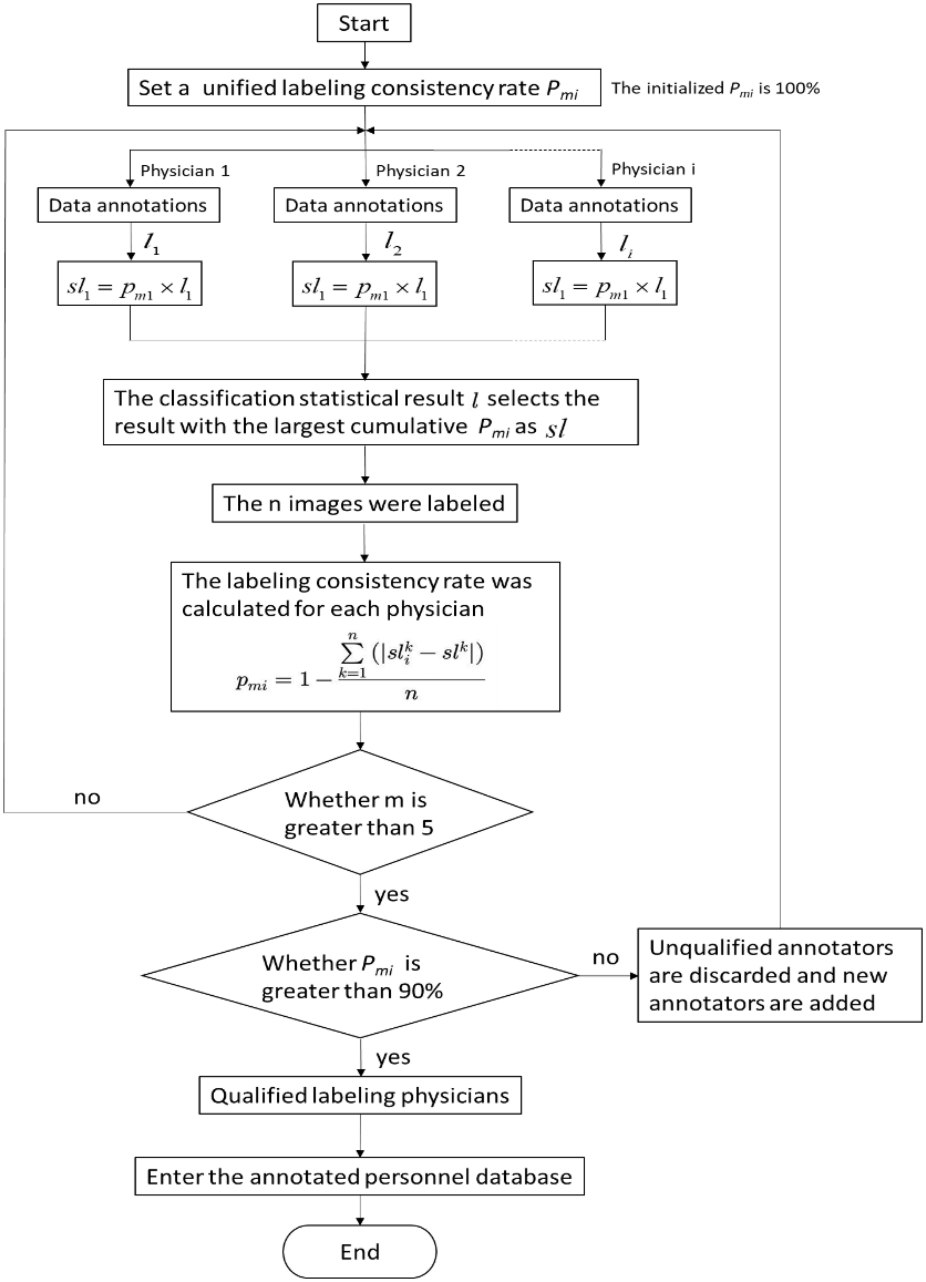
The flow chart depicting the annotation quality-control algorithm.

To eliminate inconsistency in the pneumoconiosis image data annotations, physicians used the following evaluation method consistently (Figure 1). The process considerably improved data labeling accuracy.

### 2.3 Image segmentation

**Figure 2:**
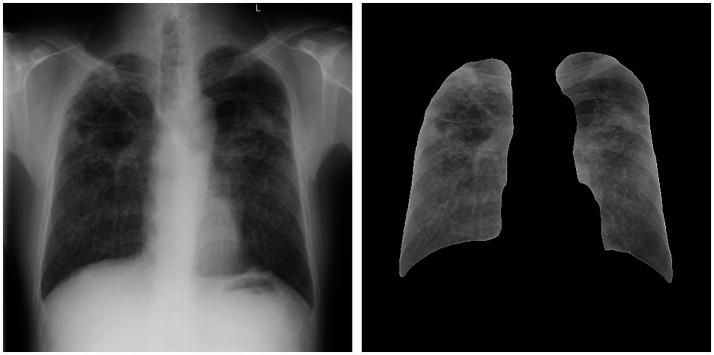
Image depicting the effect of lung field image segmentation.

The DR image segmentation model of pneumoconiosis was trained in advance on the basis of a fully convolutional network (FCN), using U-Net architecture, which was suitable for the segmentation of medical images. Figure 2 depicts the effect of lung-field image segmentation. The following steps were used to train the model:

①Data preparation: the DR image data of pneumoconiosis and the corresponding segmentation label were prepared. The segmentation label was an image with the same size as that of the DR image, and the value of each pixel indicated the category (lung or background) to which the pixel belonged. The category was manually labeled by professional doctors.
②Data preprocessing: before providing inputs to the U-Net model, DR image preprocessing and normalization were performed. Preprocessing improved network training stability as well as performance.
③U-Net model development: the model was an FCN comprising an encoder for extracting high-level characteristics of the image and a decoder for mapping these characteristics back to the original pixel space to yield segmentation results.
④U-Net model training: the model was trained with the aim of obtaining network outputs that are almost the same as the real segmentation label. This was achieved by minimizing the cross-entropy loss function.
⑤Evaluation and optimization: after model training, its performance was evaluated based on independent test data. Indices for evaluation included pixel accuracy and intersection over the union.
⑥Model application: when its performance reached the standard, the model was applied to a new DR image of pneumoconiosis for automatic lung-field segmentation.

### 2.4 DL algorithm

The CNN-based DL algorithm was trained for identifying normal and pneumoconiosis radiographs in frontal chest DR. The model was trained based on the pneumoconiosis lung-field segmentation image data and negative and positive labels. A self-developed TM-Net neural network was used to train the pneumoconiosis screening model, which was redesigned from ResNet neural network. The basic architecture of this network includes convolutional layer, pooling layer, residual block, global average pooling and fully connected layer, in which residual block is the key component. By adopting residual learning, the problem of gradient disappearance and gradient explosion in deep neural networks can be effectively solved. In terms of hyperparameters, ResNet-101 is adopted, whose model depth is 101 layers, the learning rate is set to 0.001, weight attenuation is set to 0.0001, label smoothing is set as the loss function, and data training is conducted by fixed proportion resampling method according to categories. In terms of data enhancement, DR Data of pneumoconiosis were mirrored and rotated (rotated no more than 10 degrees to simulate deviations caused by different shooting angles). A total of 49,872 patients were included in the training dataset, accounting for 80% (*n* = 39,898), whereas the test dataset accounted for 20% (*n* = 9,974) during the training process. Loss functions including label smoothing loss were used for the iterative training of the model to ensure stability and avoid the overfitting phenomenon. After model convergence, a round of data training was completed. During the iterative model training, based on model performance in the test dataset and verification results of the training dataset, data with possible problems in labeling were screened out for expert verification. After this verification, the model was trained again using the updated training dataset, until a pneumoconiosis screening model that meets the standard was trained.

The following methods were used while developing the algorithm to enhance model performance. Instead of using 8-bit PNG or JPEG files, 16-bit 512 × 512 high-resolution images from the Digital Imaging and Communications in Medicine (DICOM) datasets were used as model inputs to prevent the loss of information. These input images were normalized based on the mean ± standard deviation of the training images. The first two blocks of the model were identical and each consisted of a convolutional layer (stride = 2) and a pooling layer (stride = 2). The model output was a binary label of *y* ∈ {0, 1}, which indicated pneumoconiosis absence or presence. Focal loss and the corresponding parameters (19) were used to maintain an optimal balance between sensitivity and specificity in order to focus the training on low-prevalence difficult cases and prevent a high incidence of false negatives (FNs), thus avoiding the overestimation of the classifier during the process. Additionally, in the training process of the model, the label smoothing method is used as a loss function to balance the model performance and convergence speed (20).

More than 10 hospitals in China have installed the present DL algorithm. Thus, imaging data subsequently collected from different centers in China were used for algorithm development.

### 2.5 Model validation

The validation set comprised 495 independent high-quality labeled chest radiographs. Statistical analysis was performed using the in-house (see text footnote 1) and SPSS software (IBM SPSS Statistics, Armonk, NY). The confusion matrix, consistency, specificity, and sensitivity of the screening model were calculated. The true positive (TP, the number of patients with pneumoconiosis diagnosed as pneumoconiosis), false positive (FP, the number of normal individuals diagnosed as pneumoconiosis), true negative (TN, the number of normal individuals diagnosed as normal), FN (the number of patients with pneumoconiosis diagnosed as normal, i.e., missed cases), and sensitivity (the proportion of cases accurately diagnosed as pneumoconiosis) rates were derived for diagnostic power determination. The following formulas were used to assess algorithm performance: Specificity = TN/(FP + TN), Sensitivity = TP/(TP + FN), Precision = TP/(TP + FP), Accuracy = (TP + TN)/(TP + FP + FN + TN), and *F*1-score = 2 × Precision × Sensitivity/(Precision + Sensitivity). The classification accuracy of the model was measured using Cohen’s kappa. To determine the predictive power of the DL algorithm, a receiver operating characteristic (ROC) curve was plotted, followed by AUC calculations. Furthermore, heatmaps were generated for model visualization.

### 2.6 Computing environment

Herein, computational works were performed in a computing environment using an interface of Python 3.0 based on a Pytorch DL framework. This was installed and run on the server Ubuntu, a Linux version, 18.04 LTS in 64 bits. Ubuntu contains two Intel Xeon Skylake 6,133 processors (2.50 GHz and a 30 MB Cache), in which each processor possesses 12 cores, and there are a total of 48 logical CPU cores. This server has 320 GB RAM and eight NVIDIA Tesla v100 GPUs with 5,120 stream cores, 32 GB maximum memory, 900 GB/s maximum memory bandwidth, and 1752 Mbps memory clock speed.

## 3 Results

### 3.1 Detection of findings

A total of 49,872 radiographic images were labeled. No pneumoconiosis or other pulmonary diseases were observed in approximately 51% (25,435/49,872) of the radiographic images (normal). In 49% (24,437/49,872) of the radiographs, pneumoconiosis was detected. Among them, category-1, category-2, and category-3 pneumoconiosis accounted for 53.1% (12,967/24,437), 20.4% (4,987/24,437), and 26.5% (6,483/24,437) of the radiographs, respectively (Table 2).

**Table 2:** Pneumoconiosis categories and subcategories.

Categories	0			1			2			3		
Subcategories	0/−	0/0	0/1	1/0	1/1	1/2	2/1	2/2	2/3	3/2	3/3	3/+
*N*	25,435			12,967			4,987			6,483		
Percent (%)	51			26			10			13		

### 3.2 Effect of the resolution ratio on accuracy

The models were trained using four types of radiographs with different image formats or resolution ratios, namely JPEG-256, JPEG-512, DICOM-256, and DICOM-512, to improve model accuracy and reduce data loss from the radiographs. The accuracy for each image format was 0.87, 0.89, 0.90, and 0.95, respectively. Therefore, instead of JPEG files, high-resolution images (512 × 512) from DICOM were provided as inputs to the model, thereby minimizing data loss.

### 3.3 Evaluation of the DL algorithm

#### 3.3.1 Statistical index

**Figure 3:**
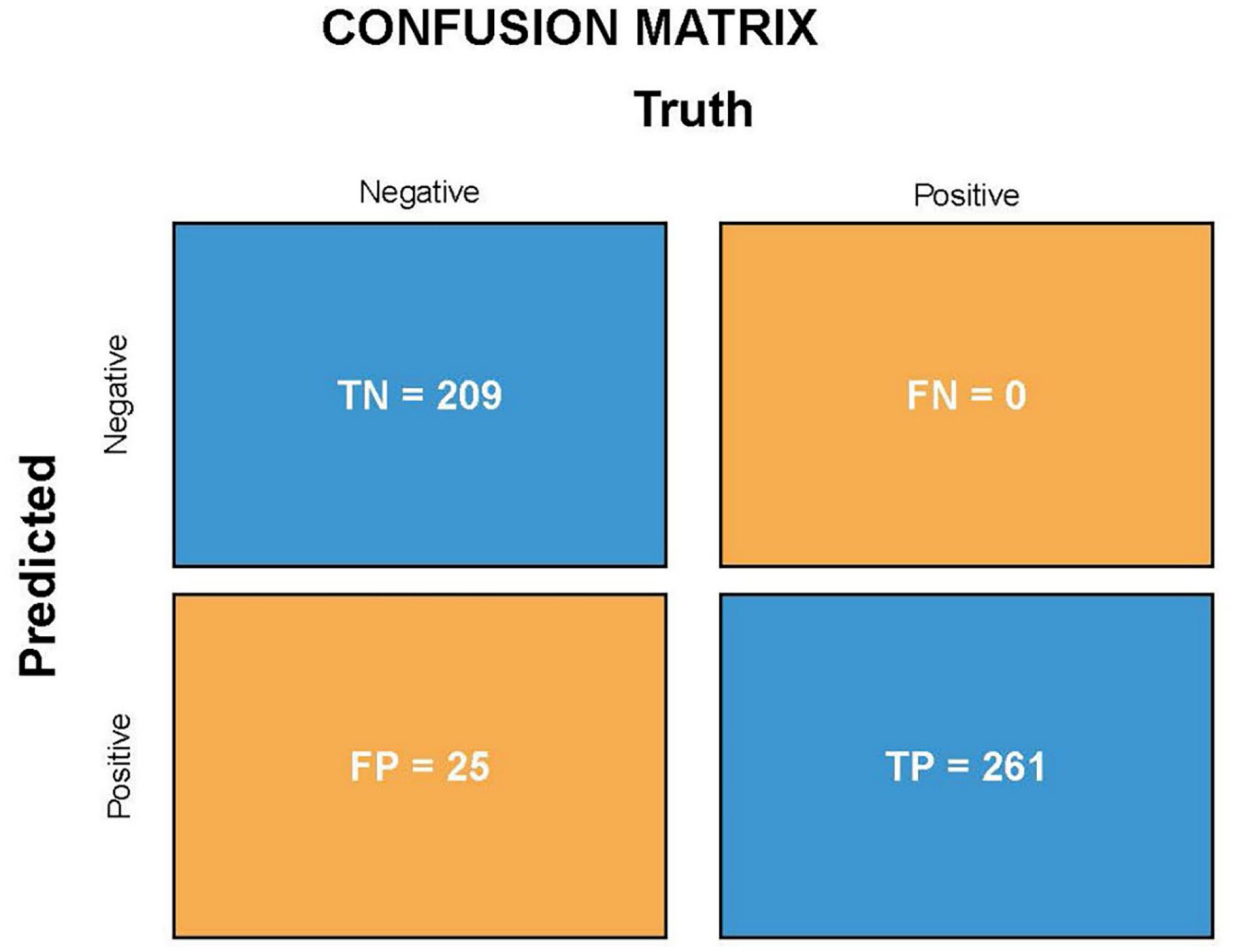
TP, FP, FN, and TN for the DL algorithm diagnosis.

Because the algorithm was developed for pneumoconiosis identification, its high sensitivity was essential to decrease the number of missed diagnoses. A resampling method was used during model training to improve model suitability and sensitivity for the physical checkup of dust-exposed workers and the screening of pneumoconiosis. The validation set comprised 495 patients with DR chest X-ray images, including 261 patients with pneumoconiosis and 234 without pneumoconiosis. The final assessment of the validation set showed that the sensitivity/recall and specificity were 100 and 89.3%, respectively, which shows the model can screen out all cases of pneumoconiosis, and the probability of missing diagnosis is very small. Moreover, the accuracy, precision and *F*1-score (*F*1-score comprehensively considers the two indexes accuracy and precision, and the closer the output is to 1, the better the model performance) were 95, 91.3% and 0.954, respectively, which means high performance for correct model diagnostics. Thirdly, Cohen’s kappa of the novel algorithm was 0.898 (~0.81–1 means perfect agreement). This indicator also indicates a high degree of agreement between AI models and expert annotations. Figure 3 shows the TP, FP, FN, and TN values of the DL algorithm.

#### 3.3.2 ROC curve

**Figure 4:**
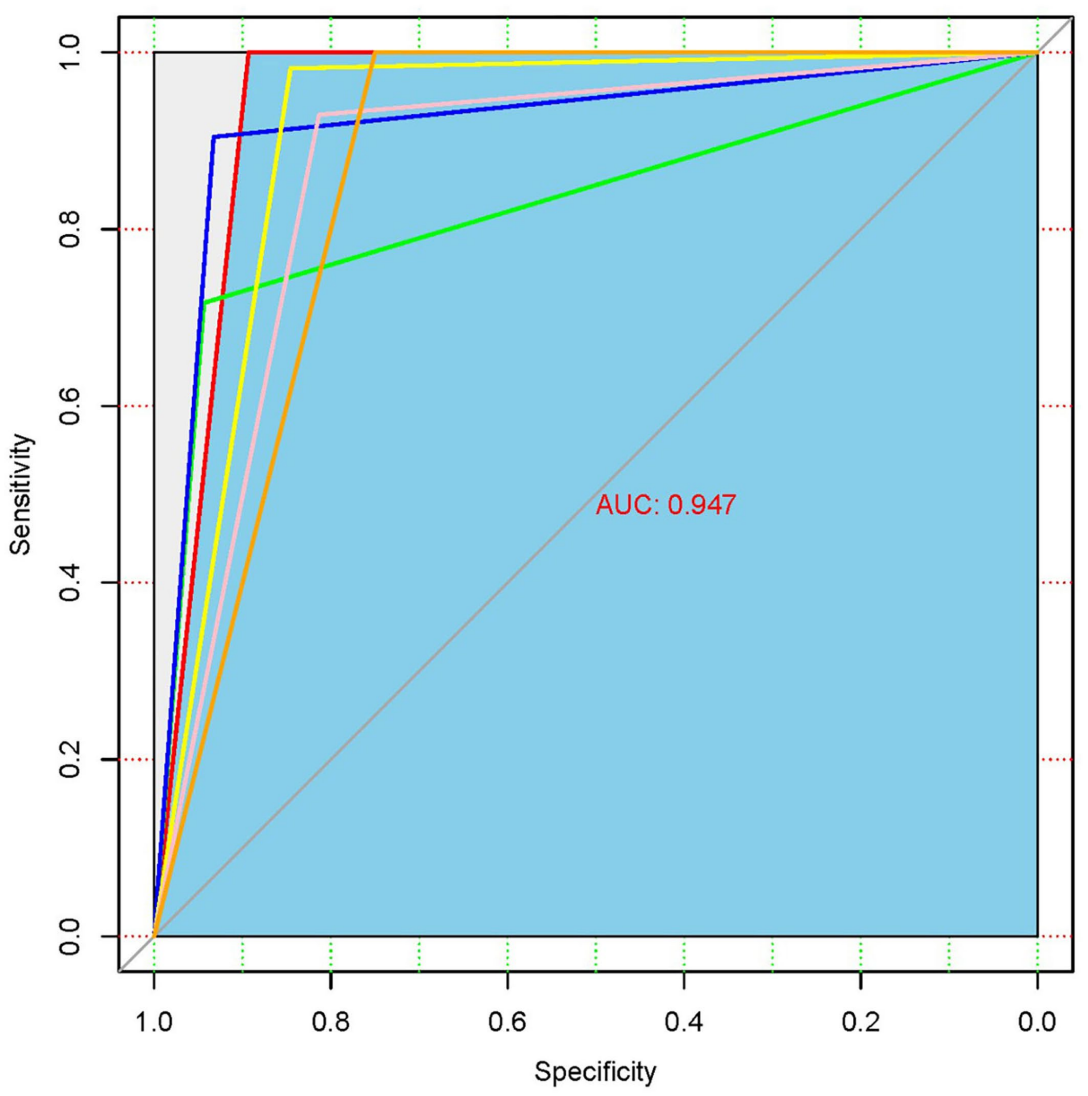
ROC for the detection of chest X-ray findings with the DL algorithm.

The ROC analysis of the chest X-ray findings using the developed DL algorithm is presented in Figure 4. AUC is an indicator that represents the overall ability of the model to distinguish real samples, and the closer it is to 1, the better the model performance. The AUC of the developed DL algorithm was 0.947, which means that the model has a high diagnostic performance for pneumoconiosis.

#### 3.3.3 Heatmaps

**Figure 5:**
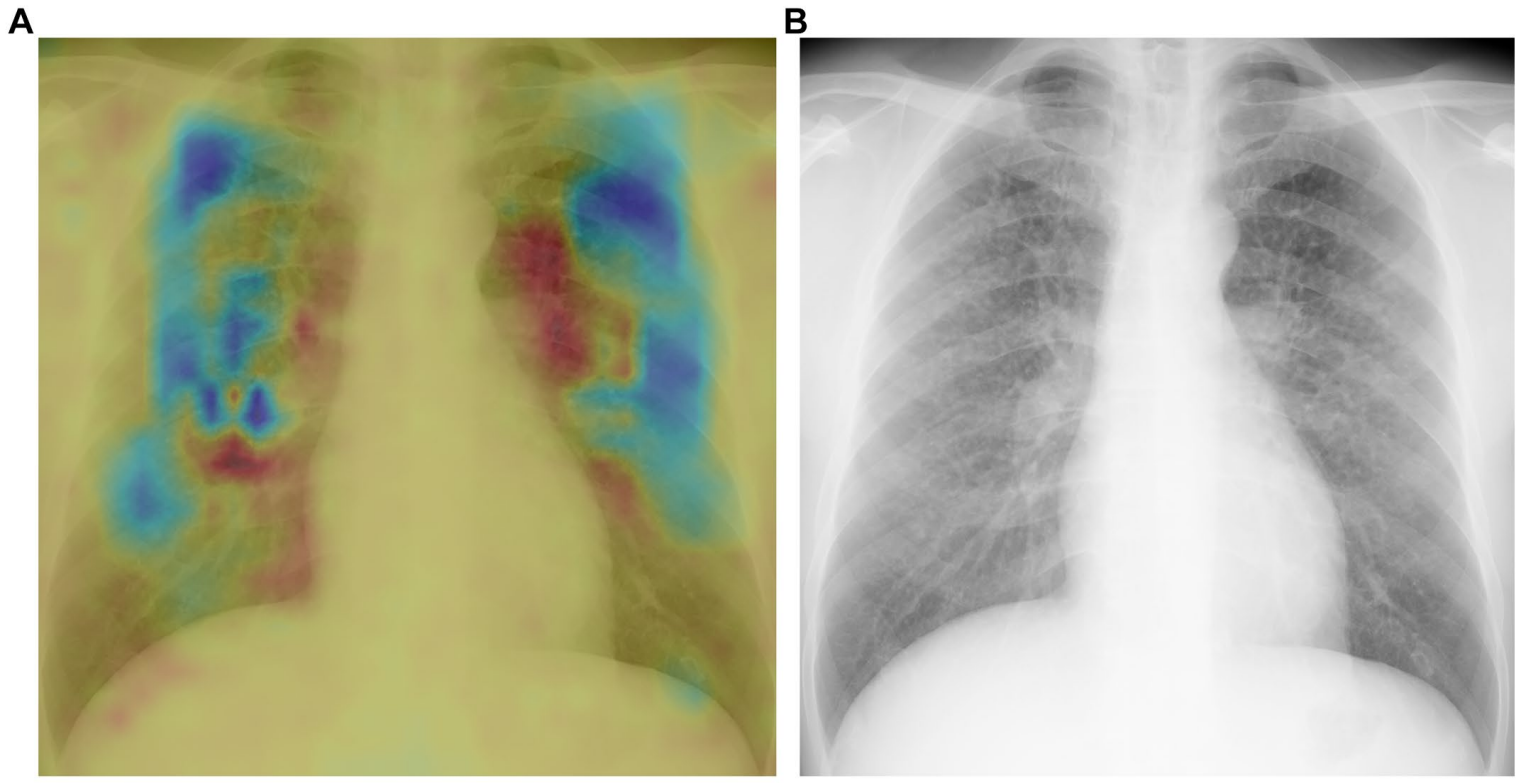
Heat maps generated by the DL algorithm.

Heatmaps generated using the novel algorithm for pneumoconiosis detection are presented in Figure 5. The red areas indicate regions that are selectively analyzed by the model, whereas the cool-colored areas are not specifically analyzed. These corresponding heatmaps (a) precisely highlight these abnormalities (b).

## 4 Discussion

In the present study, an automated model with high sensitivity (100%) and satisfactory specificity (89.3%) was developed for pneumoconiosis detection. Based on these sensitivity and specificity values, the model is suitable for screening low-prevalence pneumoconiosis cases. Because of its efficiency in identifying all potential pneumoconiosis cases, the model is ideal for screening patients with pneumoconiosis during their occupational health examination. These identified patients can be asked to consult a radiologist for image review as well as diagnosis confirmation. By using this model, radiologists’ workload can be markedly reduced without hampering diagnostic accuracy. Moreover, due to its high AUC, the present model can help reevaluate radiographic image interpretations by radiologists to avoid misdiagnoses or missed diagnoses caused by fatigue or subjectivity. Less-experienced radiologists or physicians can improve their skills in radiographic interpretations with the help of the model.

Over the years, several semi-automated and automated CAD systems for pneumoconiosis detection have been established using methods ranging from traditional image analysis to machine learning (ML) and DL approaches. Previously, CAD tools have been investigated for detecting pneumoconiosis in chest radiographs with the help of texture analysis (5). These systems were developed by manually dividing a lung field into several subdivisions, and these subdivided regions were subjected to automatic analysis. Yu et al. (6) reported a modified strategy with AUC and accuracy values of 0.948–0.978 and 87.7–89.2%, respectively. Here, a lung field showing pneumoconiosis was subdivided employing a feature-extraction method that integrated histograms generated based on multi-scale difference filtering and a gray-level co-occurrence matrix. SVM was a region-level classifier that incorporated predictions for six divisions into the final classification. Alternatively, in a study, a lung field was subdivided into six divisions, followed by segmentation by combining Otsu’s threshold method with morphological reconstruction. A computer-based method comprising 22 wavelet-based energy textures and SVM was developed for detecting abnormal regions within normal sections, showing an accuracy of 83.3% for high-opacity recognition (7). All these methods are non-neural networks; thus, they require manual feature extraction and more time. Hence, they have never been considered mainstream methods in ML. Okumura et al. (8) merged rule-based and ANN methods with three new improved techniques to differentiate abnormal regions from normal ones. The resulting diagnostic power was high for serious patients with pneumoconiosis (AUC = 0.93 ± 0.02) and moderate for early-stage patients with pneumoconiosis (AUC = 0.72 ± 0.03). In 2017, these authors made some improvements by developing a three-stage-ANN-based CAD tool, which showed satisfactory diagnostic power for serious (AUC = 0.89 ± 0.09) and early-stage (AUC = 0.84 ± 0.12) patients (9). However, being a shallow neural network, ANN cannot efficiently undergo representational learning to achieve a satisfactory accuracy value, thereby limiting its potential for high-value applications.

Recently, an increasing number of studies have focused on using a CNN model to screen patients with pneumoconiosis in DR. (21–27) Zheng et al. (21) applied the transfer learning of LeNet, AlexNet, and three versions of GoogleNet to a pneumoconiosis chest radiograph dataset for the CAD of coal workers with pneumoconiosis. Integrated GoogleNetCF outperformed the remaining models on this dataset (its accuracy was 71.6–93.88%). Hao et al. (22) used ResNet34 and DenseNet53 to train the pneumoconiosis screening model, with the accuracy of 0.893 and 0.886, respectively, and the training dataset was small, with only 142 cases. Wang et al. (23) reported inception-V3 CNN performance in pneumoconiosis chest X-ray screening, with an AUC of 0.878 [95% confidence interval (CI): 0.811–0.946]. CNN performance exceeded that of two radiologists [AUCs were 0.668 (95% CI: 0.555–0.782) and 0.772 (95% CI: 0.677–0.866), respectively]. A 2022 study showed that AED-Net was used to train a classification diagnosis model of pneumoconiosis, with accuracy and AUC values of 90.4 and 96%, respectively (24). In 2021, Devnath et al. (25, 26) proposed a novel approach for screening pneumoconiosis chest radiographs on the basis of the multi-level feature analysis of CNN architecture. The researchers applied the transfer learning obtained using CheXNet to extract multi-dimensional deep features from multiple levels of its architecture for creating a small database. Then, SVM and a CNN-based feature aggregation method were used to map deep features into high-dimensional features for classification. Further verification showed that the accuracy of the ensemble model in the automatic detection of pneumoconiosis reached 92.68%. However, the training database used was small, containing only 71 chest radiographs, which limited the improvement of its accuracy and extrapolation ability.

The present study is more advantageous than the previous studies. First, we used in-house deep CNNs for the training purpose, which included representational learning and the automatic extraction of task-related features. Moreover, compared with traditional methods, the DL approach reduces manual labor and markedly improves detection accuracy. Second, developing a reliable classifier for clinical use mostly depends on sample size and dataset quality, and accurately representing the real-world scenario is critical (28). The present dataset included 49,872 cases from China, of which 24,437 were of pneumoconiosis. As far as we know, we have reported the largest dataset in the pneumoconiosis CAD detection field. Our radiographs were obtained from various clinics and diverse patients, ranging from normal cases to abnormal ones and early-stage pneumoconiosis to serious cases. Because of the diverse and comprehensive dataset, the model was effectively subjected to various real-world clinical scenarios. Third, we included 10 experts in diagnosing occupational diseases and two professional radiologists who are authoritative in the area of pneumoconiosis to annotate the radiographs used here. Thus, we confirmed annotation result accuracy and specificity. Moreover, we applied an annotation quality-control algorithm, thus ensuring result accuracy during annotations. Finally, owing to the aforementioned efforts, the present study is more beneficial than the previous studies because the present model exhibited superior performance. Furthermore, the incidence of FNs was 0 when applied to pneumoconiosis, a low-prevalence disease, which suggested that all cases speculated as pneumoconiosis were positive for the disease. As far as we know, the present model showed the best performance among the existing models.

Despite the striking results, we need to address the study’s limitations. Pneumoconiosis diagnoses by radiologists based on radiographs are not always correct. Nevertheless, in order to minimize this deviation, we hired experienced occupational disease physicians and imaging physicians to label each chest film with 3–5 people, and ensured the correctness of diagnosis as far as possible through the procedures mentioned above (Figure 1). Moreover, long-term follow-up or further testing may be useful to confirm the diagnosis.

Taken together, the present CAD system exhibited a suitable diagnostic performance. Its performance can be improved via model refinement and using steadily larger sample sizes; thus, the CAD tool possesses the potential to detect pneumoconiosis in advance. In practical clinical work, the model is mainly used in the following scenarios: pneumoconiosis diagnosis and regular screening of dust exposed workers. The clinician or radiologist can use this model to pre-screen a large number of chest radiographs, and then only perform manual interpretation of positive chest radiographs, which reduces workload and improves work efficiency. Or for chest radiographs interpreted as negative by radiographers, CAD model is used for secondary interpretation to reduce the occurrence of missed diagnosis and misdiagnosis. This model will play an important role in clinical work as an auxiliary tool for manual film reading. As more and more chest radiographs with multiple confirmations are added to the database, the performance of our AI models will become better and better, even better than that of most imaging physicians, and at that time, AI models may become the gold standard of diagnosis rather than just a manual aid. Moreover, we aim to use the reported database to continuously develop more DL algorithms for diagnosing thoracic diseases, and these algorithms will be clinically useful.

## 5 Conclusion

Based on CNN algorithm and large-scale accurately labeled pneumoconiosis chest X-ray database, an AI model has been trained for pneumoconiosis screening. With ultra-high sensitivity and excellent performance, it can help radiologists improve the efficiency and accuracy of pneumoconiosis diagnosis, and has broad application space.

## Data availability statement

The original data supporting the conclusions of this paper are non-public and can be obtained conditionally.

## Ethics statement

The studies involving humans were approved by the Medical Science Research Ethics Committee of Peking University Third Hospital [Ethics No. (2023) Medical Ethics Review No. (100-01)]. The studies were conducted in accordance with the local legislation and institutional requirements. Written informed consent was obtained from the individual(s) for the publication of any potentially identifiable images or data included in this article.

## Author contributions

XL: Formal analysis, Funding acquisition, Methodology, Writing – original draft, Writing – review & editing, Conceptualization, Data curation, Investigation, Project administration. MX: Methodology, Software, Writing – review & editing. ZY: Methodology, Software, Writing – review & editing. FX: Methodology, Software, Writing – review & editing. SL: Conceptualization, Supervision, Validation, Writing – review & editing. YZ: Supervision, Validation, Writing – review & editing. ZX: Investigation, Writing – review & editing. LG: Conceptualization, Supervision, Validation, Writing – review & editing.

## Figures and Tables

**Table:** 

DL	Deep learning
AI	Artificial intelligence
CNN	Convolutional neural network
ILO	International Labor Organization
CAD	Computer-aided diagnosis
ANN	Artificial neural network
SVM	Support vector machine
AUC	Area under the curve
DR	Digital radiography
FCN	Fully convolutional network
DICOM	Digital Imaging and Communications in Medicine
FN	False negative
TP	True positive
FP	False positive
TN	True negative
ROC	Receiver operating characteristic
ML	Machine learning

## Footnotes

^1^http://anno.maxdiag.com/
